# Modeling of Heat Stress in Sows Part 2: Comparison of Various Thermal Comfort Indices

**DOI:** 10.3390/ani11061498

**Published:** 2021-05-21

**Authors:** Mengbing Cao, Chao Zong, Yanrong Zhuang, Guanghui Teng, Shengnan Zhou, Ting Yang

**Affiliations:** 1 College of Water Resources and Civil Engineering, China Agricultural University, Beijing 100083, China; mengbing-cao@cau.edu.cn (M.C.); zyr123@cau.edu.cn (Y.Z.); zhoushengnan@cau.edu.cn (S.Z.); sy20193091754@cau.edu.cn (T.Y.); 2 Key Laboratory of Agricultural Engineering in Structure and Environment, Ministry of Agriculture and Rural Affairs, Beijing 100083, China

**Keywords:** temperature and humidity index, black globe-humidity index, effective temperature, equivalent temperature index for sows, enthalpy

## Abstract

**Simple Summary:**

Various thermal indices have been developed to evaluate the heat stress in animals. In this study, the temperature and humidity index (THI), effective temperature (ET), enthalpy (H), and the equivalent temperature index of sows (ETIS) from the part 1 of this paper series have been reviewed and analyzed in the process of sow production. Four approaches have been proposed to analyze these commonly applied thermal indices: (1) equivalent temperature change method; (2) the method based on the change of thermal index under different wind speeds; (3) the psychrometric chart method; and (4) CFD simulation method. In the analysis among those thermal indices, the ETIS performed best in evaluating the sow’s thermal environment, followed by THI2, THI4 and THI7. This research provides a theoretical basis for selecting an appropriate thermal index for thermal environment evaluations in the sow production.

**Abstract:**

Heat stress has an adverse effect on the production performance of sows, and causes a large economic loss every year. The thermal environment index is an important indicator for evaluating the level of heat stress in animals. Many thermal indices have been used to analyze the environment of the pig house, including temperature and humidity index (THI), effective temperature (ET), equivalent temperature index of sows (ETIS), and enthalpy (H), among others. Different heat indices have different characteristics, and it is necessary to analyze and compare the characteristics of heat indices to select a relatively suitable heat index for specific application. This article reviews the thermal environment indices used in the process of sow breeding, and compares various heat indices in four ways: (1) Holding the value of the thermal index constant and analyzing the equivalent temperature changes caused by the relative humidity. (2) Analyzing the variations of ET and ETIS caused by changes in air velocity. (3) Conducting a comparative analysis of a variety of isothermal lines fitted to the psychrometric chart. (4) Analyzing the distributions of various heat index values inside the sow barn and the correlation between various heat indices and sow heat dissipation with the use of computational fluid dynamics (CFD) technology. The results show that the ETIS performs better than other thermal indices in the analysis of sows’ thermal environment, followed by THI2, THI4, and THI7. Different pigs have different heat transfer characteristics and different adaptability to the environment. Therefore, based on the above results, the following suggestions have been given: The thermal index thresholds need to be divided based on the adaptability of pigs to the environment at different growth stages and the different climates in different regions. An appropriate threshold for a thermal index can provide a theoretical basis for the environmental control of the pig house.

## 1. Introduction

High temperatures reduce a sow’s estrus [1] and pregnancy rate [2,3], decrease milk production [4], cause weight loss during lactation [5], and increase mortality [6]. As the core driving force in pig farm production, sow production directly affects the number and quality of piglets, and further influences the overall performance of a farm. According to reports, heat stress can cause hundreds of millions of dollars in losses to pig farms [7]. With the advent of global warming, the heat stress issue on pig farms is receiving more attention [8].

Reasonable quantification of heat stress can provide guidance for effectively regulating the thermal environment of the pig house. The commonly used thermal indices are temperature and humidity index (THI) [9,10,11,12,13,14,15,16], the globe-humidity index (BGHI) [17,18], effective temperature (ET) [19], equivalent temperature index of sows (ETIS) [20], and enthalpy (H) [21]. Among them, THI has many varieties derived from the original, and eight types of THI index have commonly been used:

(1) THI1 was developed by Thorn [9] and used as a human comfort index by the US Meteorological Administration [17]. Sales et al. [22] used the THI1 environmental index to analyze the impact of thermal environment on the reproductive performance of sows. The thresholds of THI1 were divided as follows: heat comfort zone of 61 < THI1 ≤ 65, mild heat stress of 65 < THI1 ≤ 69, and heat stress zone of 69 < THI1 ≤ 73.
(2) The thermal index THI2, developed by Thom [10], was also for human comfort investigations. Later, the index was also used to determine the degree of heat stress in cattle and pigs. Mader et al. [23,24] used THI2 to analyze the heat stress in cattle, while Vashi et al. [25] used it to analyze the changes of different hormones in pigs during different seasons to determine the adaptability of pigs to the environmental changes of different seasons. Godyn et al. [26] used THI2 to study the effect of the atomization system on the microclimate of the farrowing room and the effect this had on sow welfare. The respiratory rates and rectal and skin surface temperatures of sows in different environments were analyzed. Yosuke et al. [27] also studied the effects of THI2 and maximum temperature on the farrowing rate of sows. Mellado et al. [28] used THI2 to analyze the relationship between THI2 and the reproductive performance of sows. When THI2 < 74, the pregnancy rate was 93%, and as THI2 further decreased, the pregnancy rate continued to increase. When 74 ≤ THI2 < 78, the pregnancy rate was 91.8%, and the pregnancy rate in this interval was relatively stable. When 78 ≤ THI2 < 82, the pregnancy rate was 91.4%, which was lower than before, and the pregnancy rate was relatively stable. When THI2 > 82, the pregnancy rate of sows was 89.8%. When THI2 was lower than 82, the pregnancy rate continued to decline. Therefore, according to the research of Mellado et al., THI2 can be classified into certain heat stress thresholds: THI2 < 74 indicates comfortable environment, 74 ≤ THI2 < 78 indicates mild heat stress, 78 ≤ THI2 < 82 indicates moderate heat stress, and THI2 ≥ 82 indicates severe heat stress.
(3) Ingram [11] determined the weight of wet bulb temperature using THI3 according to the degree of influence of relative humidity in the air on pigs. Wang [29] analyzed the correlation between the THI3 index and the behavioral physiological response of pigs. Studies have shown that when THI3 is greater than 28, heat stress responses, such as increased body temperature, will occur.
(4) Kelly et al. [12] proposed THI4 in their literature. Tummaruk et al. [30] used high temperature, high relative humidity and high THI4 to analyze the impact of the thermal environment on a sow’s litter size. Pu [31] used this index to analyze the effects of thermal environment on pig feeding behavior and pig physiological indicators. However, the thermal threshold division of this index is still unclear.
(5) Maust et al. [13] used the index THI5 to analyze the effect of the comprehensive thermal environment on the performance of dairy cows during lactation. Lucas et al. [32] used THI5 to analyze the impact of the thermal environment on pigs and proposed that an evaporative cooling system could be a feasible and economical solution to the heat stress in pigs. When the index value reached 75, it indicated a heat stress level, and a value of 83 was a dangerously high level [33].
(6) The National Weather Service Central Region (NWSCR) [16] of the United States proposed THI6, and a large number of scholars have used this index to analyze the impact of climate on the reproductive performance of sows. The threshold zone of the index [34] is: THI6 ≤ 74 means suitable environmental level, 74 < THI6 ≤ 78 means mild heat stress level, 78 < THI ≤ 84 means moderate heat stress level, THI > 84 means severe heat stress level.
(7) The National Oceanic and Atmospheric Administration (NOAA, 1976) of the United States [14] proposed THI7. Iida et al. [35] used this index to analyze the reproductive performance of sows and determine the impact of climate on sow production, but the index did not have a threshold division.
(8) Fehr et al. [15] used THI8 to analyze the effect of evaporative cooling on pigs, and used evaporative cooling to reduce the heat stress level in pigs, but the index did not have a good threshold division.

Aside from THI indices, the black globe-humidity index (BGHI) is another type of thermal index commonly applied in animal production, which is also a variant of THI1. Buffington et al. [17] used the THI1 index for analysis of the environment of cattle, and the dry bulb temperature in THI1 was replaced by the black globe temperature to reveal the effect of radiative heat transfer. Junior et al. [18] used BGHI to study the effect of high temperature environments on sow lactation rate in a conventional environment, a floor cooling environment, and a semi-outdoor environment.

The effective temperature (ET) was used by Beckett [36] to evaluate environmental conditions, and humidity and air velocity were integrated into this index to study the impact of the environment on animals. Beckett provided a chart to illustrate the combined effect of air temperature and humidity on pig growth. Bjarne et al. [19] used the research of Beckett [36], Ingram [11], and Roller et al. [37] to determine the relative humidity item of ET. It was supposed that when the air temperature got close to the temperature of the pig body, the cooling effect achieved by the ventilation would be gradually weakened. It was also assumed that the cooling effect was proportional to the power function of air velocity, and the power function of the cooling effect and air velocity was determined according to the relationship between air velocity and pig convective heat transfer coefficient studied by Li et al. [38]. Finally, ET was expressed through the terms of ambient temperature, relative humidity, and air velocity. Bjarne et al. analyzed the correlation between ET and pig heat flux, and the ET performed well in pigs weighing between 3.4 kg and 70 kg.

The equivalent temperature index of sows (ETIS) was established in the part 1 of this paper series [20], which looked at the combined effect of the heat transfer characteristics of sows, integrated air temperature, relative humidity, and air velocity on physiological characteristics of sows. Based on the correlation between ETIS and THI2, ETIS was divided into several thresholds. The ETIS was mainly used to assess the heat stress in sows.

Enthalpy is an important state parameter of the energy of the material system [39,40], and enthalpy has often been used together with the psychrometric chart when describing environmental conditions. Enthalpy includes the influence of temperature and relative humidity in the air, so it can also be used as an index to evaluate the environment. Rodrigues et al. [21] extended the enthalpy equation to a certain extent, and finally determined the enthalpy value as the equation H, which was used as the comfort index of livestock and poultry.

The above mentioned heat stress indices have all been used to assess the heat stress in sows, but most heat indices are not developed based on sows [41,42], of which ET is used to analyze pigs from 3.4 kg to 70 kg, and only ETIS was developed for sows. The heat stress thresholds of different heat indices are also different. Using different methods for establishing a thermal index will also lead to different evaluation effects [41,42]. Therefore, for different environments, the applied thermal index should be evaluated for better performance. In order to avoid improper use of the heat index, a comprehensive understanding and comparison of the heat indices should be carried out [41].

The heat index usually mainly includes temperature, relative humidity and air velocity. The air temperature is the dominant factor of the overall thermal environment. Relative humidity is an important factor affecting the thermal environment. Whether the change of relative humidity has a reasonable influence on the thermal index is usually analyzed by the equivalent temperature method [41,43]. Air velocity is also an important factor affecting the thermal environment, as the air velocity directly affects the convective heat transfer of pigs. The influence of air velocity on the thermal index requires further analysis. The psychrometric chart has often been used to compare the variation trend of different heat indices. Meanwhile, the heat indices have also been combined with CFD simulations [19,44] to analyze the environmental distributions in the pig house. Therefore, the correlation between the heat index around the sow at different locations and the heat transfer of the sow can be used as a method in assessing the applicability of the heat indices.

Therefore, this study uses the following methods to analyze various thermal indices: (1) The equivalent temperature change method; (2) variation trend of heat indices under different air velocities; (3) psychrometric charts; and (4) CFD simulation. This study provides a reference for the selection of a heat index to analyze a sow’s environment. A reasonable heat index is a powerful basis for regulating and optimizing the environment.

## 2. Materials and Methods

### 2.1. Various Thermal Indices

When the air temperature is kept constant, different relative humidity will make animals have different heat stress responses, mainly because high humidity will inhibit latent heat dissipation. Different air velocity will make animals have different heat stress responses, mainly because the air velocity directly affects the convective heat transfer of animals. The expression of environmental conditions requires comprehensive consideration of multiple environmental factors, so the temperature and humidity index (THI) [9,10,11,12,13,14,15,16], black globe-humidity index (BGHI) [17,18], effective temperature (ET) [19], equivalent temperature index of sows (ETIS) [20], and enthalpy (H) [21] are often used to analyze the environment of the sow house. The equations for different thermal indices are shown in Table 1, and only some of the thermal indices have thresholds divisions.

### 2.2. Definition of Equivalent Air Temperature Change

In order to analyze the performance of each thermal index in evaluating the environmental thermal effect, this study uses a comparison method called the equivalent temperature change to analyze the change of the thermal index due to humidity changes. To calculate the equivalent air temperature (T_equ_) change of the other parameters, e.g., relative humidity changes, the heat index is held constant in the calculating process. Taking the change of relative humidity from 50% to 60% as an example, as shown in Equation (1), when the temperature T_1_ is 25 °C, the relative humidity RH_1_ is 50%, and 60% is RH_2_. In order to ensure that THI remains constant before and after environmental changes, T_2_ has been adjusted. The equivalent temperature change corresponding to this process is shown in Equation (2). The air temperatures selected are 25 °C, 30 °C, 35 °C, and 40 °C, and the relative humidity changes from 50% to 60%, and the corresponding equivalent temperature changes of the thermal index in different environments have been calculated [41]. The ET and ETIS indices contain the parameter of air velocity, so when comparing with other temperature and humidity indices, the air velocity in ET and ETIS is determined to be 1 m·s^−1^. The dew point temperature contained in other heat indices can be transformed by Equation (3) [45], the wet bulb temperature can be transformed by Equation (4) [46], and the black bulb temperature can be transformed by Equation (5) [47] to the air temperatures. The air temperature using degrees Fahrenheit involved in the heat index is transformed by Equation (6) to degrees Celsius.

(1) $$\mathrm{THI}\left(T_{1},{\mathrm{RH}}_{1}\right)=\mathrm{THI}\left(T_{2},{\mathrm{RH}}_{2}\right)$$

(2) $$T_{\mathrm{equ}}=T_{1}-T_{2}$$

(3) $$T_{\mathrm{dp}}=\left(0.198+0.0017\cdot T\right)\cdot \mathrm{RH}+0.84\cdot T-19.2$$

(4) $$T_{\mathrm{wb}}=\frac{-5.86154+0.58174\cdot T+0.1485\cdot \mathrm{RH}-0.00191\cdot {\mathrm{RH}}^{2}+1.01768\cdot 10^{-5}\cdot {\mathrm{RH}}^{3}}{1+0.0036\cdot T-9.79822\cdot 10^{-5}\cdot T^{2}+9.26824\cdot 10^{-7}\cdot T^{3}-0.00899\cdot \mathrm{RH}+4.38111\cdot 10^{-5}\cdot {\mathrm{RH}}^{2}}$$

(5) $$T_{g}=0.567\cdot T+0.393\cdot \left[\frac{\mathrm{RH}}{100}\cdot 6.105\cdot \exp\left(\frac{17.27\cdot T}{237.7+T}\right)\right]+3.94$$

(6) T°=1.8·T+32

where T_1_ is air temperature of 25 °C, 30 °C, 35 °C, or 40 °C. RH_1_ is relative humidity of 50%. RH_2_ is relative humidity of 60%. T_2_ is the air temperature (°C) calculated by Equation (1). T_equ_ is the air temperature (°C) difference between T_1_ and T_2_. T is the dry bulb temperature (°C). T° is the dry bulb temperature in Fahrenheit degree (°F). T_g_ is the black globe temperature (°C). T_wb_ is the wet bulb temperature (°C). T_dp_ is the dew point temperature (°C). RH is the relative humidity (%).

### 2.3. The Effect of Air Velocity on Effective Temperature and Equivalent Temperature Index of Sows

In order to determine the changes of ET and ETIS under different air velocities, the air temperature is set to 25 °C, 30 °C, 35 °C, and 40 °C, the relative humidity to 60%, and the air velocity is changed from 0 m·s^−1^ to 4 m·s^−1^ [43].

When the thermal environment is winter, assuming a temperature of 10 °C, a relative humidity of 60%, and an air velocity of 0 m·s^−1^, ET and ETIS are 21.2 °C (ET_winter_) and 18.6 °C (ETIS_winter_), respectively. With the increasing airflow velocity, the intersection points of the ET and ETIS curves with ET_winter_ and ETIS_winter_ are analyzed.

### 2.4. Psychrometric Chart

A psychrometric chart has often been used to determine the temperature and humidity conditions in livestock and poultry houses under different environmental conditions. The heat index contains the influence of temperature and humidity in the psychrometric chart, so each heat index can be looked up in the psychrometric chart to make comparisons. Taking the temperature of 22 °C and the relative humidity of 70% as the standard value for all the heat indices, keeping each heat index constant, when the temperature and relative humidity change, the constant-index line in the psychrometric chart can be drawn. The airflow velocity of ET and ETIS was set to 0 m·s^−1^, as the applied minimum ventilation and the blockage between sows make the air speed near the sow quite low.

### 2.5. Computational Fluid Dynamics Analysis

The CFD model in this investigation is simplified by having a pig house with an air inlet at one end and a mechanical exhaust on the other. There are a total of 10 sows in the pig house. It is assumed that the inlet temperature of 30 °C, the relative humidity of 60%, the sow body temperature of 38 °C, and the exhaust port velocity of 1 m·s^−1^ are used as boundary conditions, and the distributions of heat indices in the pig house are simulated by the CFD method. The maximum values of all contours using different thermal indices are a temperature of 34 °C, a relative humidity of 60%, and an air speed of 0 m·s^−1^, and the minimum values are a temperature of 30 °C, a relative humidity of 60%, and an air speed of 1 m·s^−1^. The average heat index value is calculated from the values at four points around the sow (up, down, left, and right, 0.1 m away from the sow). The correlation between the average heat index around sows at different locations and the convective heat transfer of the corresponding sows are analyzed.

#### 2.5.1. Geometric Model

**Figure 1:**
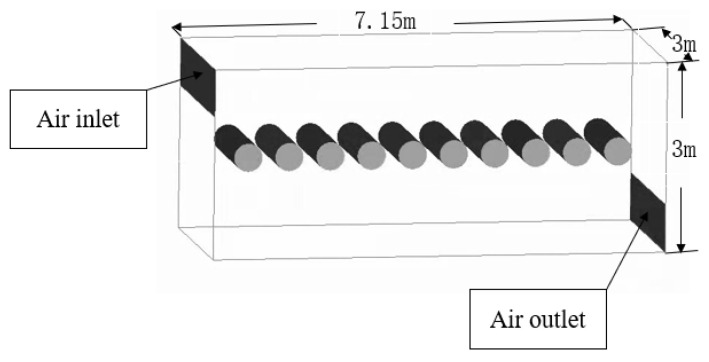
Computational fluid dynamics geometric model. The distance between the sows is 0.65 m. The length and width of the air inlet and air outlet are both 3 × 0.75 m.

The geometric model is a simplified pig house. The length, width, and height of the pig house are 7.15 m × 3 m × 3 m, respectively. 10 cylinders with a diameter-to-length ratio of of 1:4 [38] are used to represent those sows. Assuming the sow is 200 kg, the area of the sow is 2.775 m^2^ [48,49]. To ensure that the environment inside the pig house is distinguishable, and to analyze the influence of different environments on the heat transfer of sows, the position of the air inlet is set higher than that of the sows, and the position of the air outlet is set lower than that of the sows. The details are shown in Figure 1.

#### 2.5.2. Grid and Grid Independence Test

In this study, unstructured grids were used around the sow body, and structured grids were used in other areas. In order to ensure the boundary layer of the sow body [38,50], grid Y+ is kept less than 1. In this study, the first layer of grid is set to 0.07 mm. In order to minimize the influence of grid thickness, it is necessary to check the grid independence. Three resolutions of grids are built, namely fine grid (5, 139, 749), medium grid (4, 302, 437), and coarse grid (3, 016, 796). During simulation, the air velocity at the exhaust outlet is 1 m·s^−1^, the inlet temperature is 30 °C, and the sow body temperature is 38 °C. The convective heat transfer of sows in the three grid resolutions have been calculated, and the values are 1576.63 W, 1583.02 W, and 1567.69 W for fine, medium, and coarse grid, respectively. The relative difference of the convective heat transfer coefficient between the cases of fine and medium grids is 0.4%, which indicates that the medium grid is sufficient to achieve grid convergence.

#### 2.5.3. Boundary Condition Setting and Solution

The settings of boundary conditions are shown in Table 2. All simulations use the standard k-ε turbulence model [51]. The second-order precision SIMPLE algorithm has been chosen. Continuity, velocity, energy, turbulent kinetic energy (k), turbulent energy dissipation rate (ε), absolute residuals of heat transfer on the surface of sows, and absolute residuals of net flow at the inlet and outlet in the computational domain are monitored. When the heat flux on the body surface of the sow is less than 0.01% in 50 iterations and the net mass flow between the inlet and outlet is less than 10^−4^ kg∙s^−1^, the iteration is considered to be convergent.

## 3. Results

### 3.1. Comparison Using Equivalent Temperature Change Method

**Figure 2:**
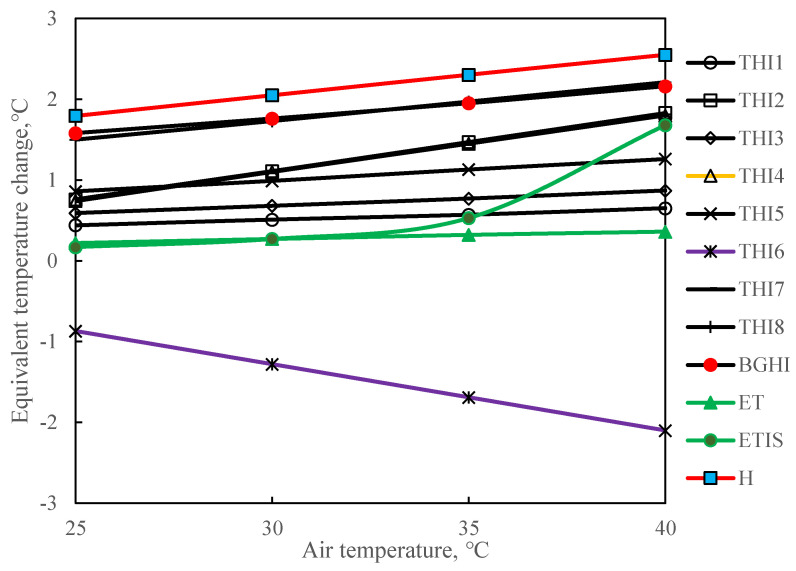
Equivalent temperature change. To calculate the equivalent air temperature (T_equ_) change of the other parameters, e.g., relative humidity changes, the heat index is held constant in the calculating process. In this comparison, the air velocity of effective temperature and equivalent temperature index of sows is set as 1 m∙s^−1^. THI is the temperature and humidity index. The numbers 1–8 after THI represent different forms of THI, which are shown in Table 1. BGHI is black globe-humidity index. Et is effective temperature, ETIS is equivalent temperature index of sows. H is enthalpy.

The equivalent temperature change method is used to compare the 12 environmental thermal indices. Figure 2 shows the equivalent temperature change of the relative humidity rising from 50% to 60% at temperatures of 25 °C, 30 °C, 35 °C, and 40 °C. It can be seen from Figure 2 that, except for THI6, the equivalent temperatures of other thermal indices are all positive. At different air temperatures, the equivalent temperature changes of THI1, THI3, THI5, and ET caused by changes in relative humidity are different, but the changing trends are nearly parallel to each other. The change trends of THI2, THI4, and THI7 are similar. For H, THI8, and BGHI at an air temperature of 25 °C, the change of 10% relative humidity causes an equivalent temperature of more than 1.5 °C. For ETIS, the equivalent temperature change becomes larger as the air temperature rises from 25 °C to 40 °C.

### 3.2. The Effect of Air Velocity

**Figure 3:**
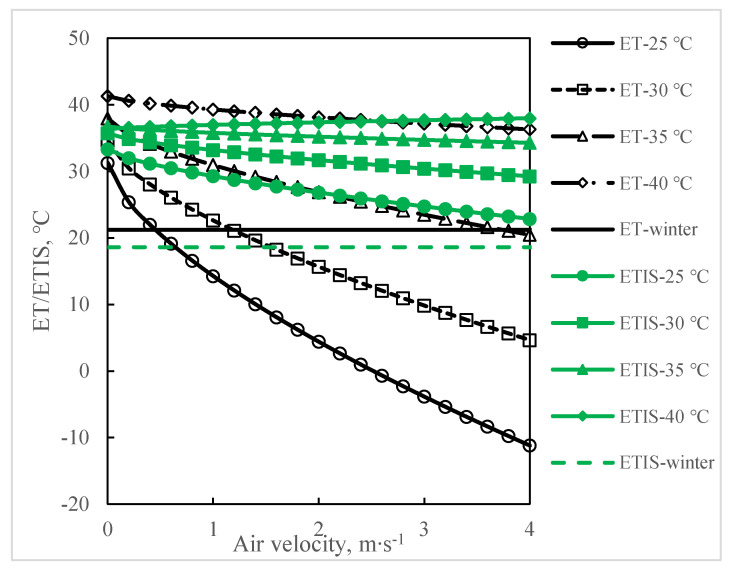
The change of effective temperature or equivalent temperature index of sows with the change of air velocity. When the relative air humidity is 60% and the air temperature is 20 °C, 30 °C, 35 °C, and 40 °C, the air velocity increases from 0 m∙s^−1^ to 4 m∙s^−1^ to analyze the changes in ET and ETIS. ET_winter_ and ETIS_winter_ represent a winter climate with an air temperature of 10 °C, a relative humidity of 60%, and an air velocity of 0 m∙s^−1^(ET_winter_ = 21.2 °C, ETIS_winter_ = 18.6 °C). With the increase of airflow velocity, a chilling effect will occur, and both ET and ETIS will decrease with the increasing air velocity. When the ET has been applied, there will be interlaces between ET_winter_ and ET of 25 °C, 30 °C, and 35 °C, which does not match the real conditions. In this situation, the ET performs inaccurately predicts the thermal conditions in the sow barn.

Figure 3 shows the change of ET/ETIS with the change of air velocity. When the relative humidity is 60% and the air temperature is 20 °C, 30 °C, 35 °C, and 40 °C, the air velocity increases from 0 m∙s^−1^ to 4 m∙s^−1^ to analyze the changes in ET and ETIS. As the air velocity increases, ET curves show downward trends. When the ambient temperature is 25 °C and the air velocity increases from 0.4 m∙s^−1^–0.5 m∙s^−1^, the ET_25 °C_ line intersects the ET_winter_ line. When the ambient temperature is 30 °C, the intersects between the ET_30 °C_ line and ET_winter_ line occurs at a velocity of 1.1 m∙s^−1^–1.2 m∙s^−1^. For the ambient temperatures of 20 °C, 30 °C, and 35 °C, as the air velocity increases, ETIS curves also show downward trends, but those curves do not intersect with the ETIS_winter_ line. For the ambient temperature of 40 °C, as the air velocity increases, ETIS shows an upward trend.

### 3.3. Comparison Using the Psychrometric Chart

**Figure 4:**
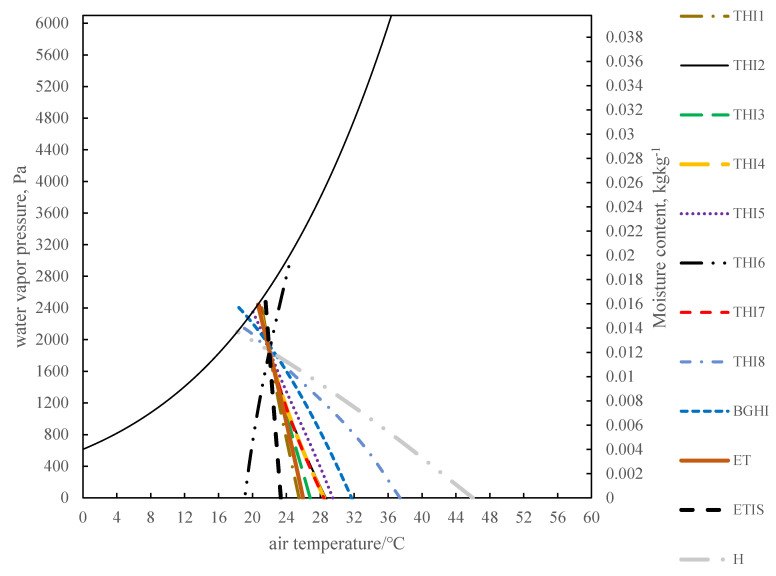
The patterns of the thermal indices in the psychrometric chart. All heat indices are consistent with the heat index value when the air temperature is 22 °C and the relative humidity is 70%. In this way, the iso-index lines of various heat indices are drawn. When compared with other thermal indices, the air velocity of effective temperature and equivalent temperature index of sows is taken as 0 m∙s^−1^. THI is the temperature and humidity index. The numbers 1–8 after THI represent different forms of THI, which are shown in Table 1. BGHI is black globe-humidity index. ET is effective temperature, ETIS is equivalent temperature index of sows. H is enthalpy.

Figure 4 shows the thermal indices fitted on the psychrometric chart. The heat index value of THI6 at an air temperature of 25.4 °C and a relative humidity of 100% is consistent with the heat index value of an air temperature of 22 °C and a relative humidity of 70%. When the air temperatures are 46 °C, 37.41 °C, and 31.6 °C for H, THI8, and BGHI, respectively, under a relative humidity of 0%, the calculated H, THI8, and BGHI are the same as those of H, THI8, and BGHI with an air temperature of 22 °C and a relative humidity of 70%. The results of THI2, THI4, and THI7 are similar. For the relative humidity changes from 0% to 70%, assuming the THI2 constant, the air temperature will change from 22 °C to 28.4 °C. In THI5, the temperature varies greatly under different humidities. There is little difference in the performance between THI1, THI3, and ET. The heat index value of ETIS at an air temperature of 23.34 °C and a relative humidity of 100% is the same as the value under an air temperature of 22 °C and a relative humidity of 70%. It is important to note that the ETIS curve and the constant-temperature line in the psychrometric chart are different.

### 3.4. Comparison Using Computational Fluid Dynamics Methods

**Figure 5:**
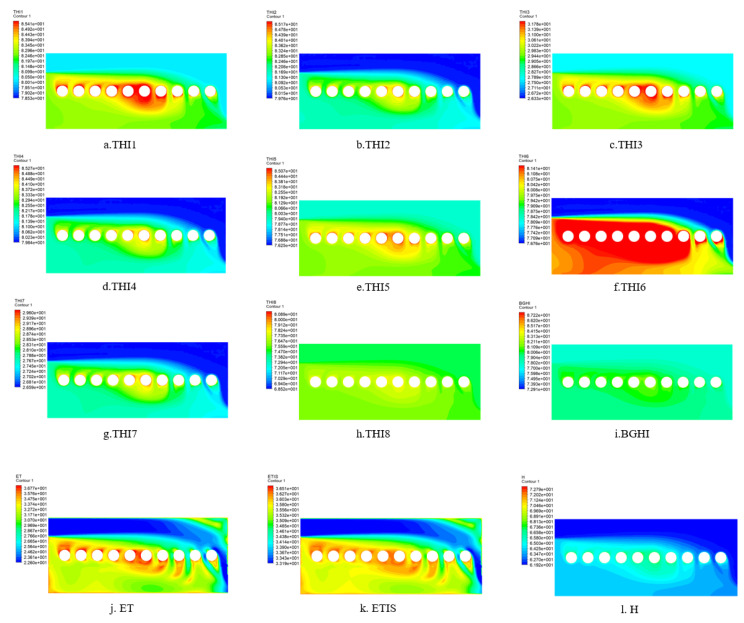
The distribution of various heat indices in the cross section of the pig barn. THI is the temperature and humidity index. The numbers 1–8 after THI represent different forms of THI, which are shown in Table 1. BGHI is black globe-humidity index. ET is effective temperature. ETIS is equivalent temperature index of sows. H is enthalpy.

Figure 5 shows the distributions of the thermal index values. When using THI2, THI4, and THI7 to analyze the thermal conditions at different zones in the pig barn, it has been found that the patterns of all the three indices are the same, with a lower index value at the air inlet area and a higher value near the animal occupied zone, which indicates that there is a difference of thermal distribution in the pig house. The distribution patterns of THI1, THI3, and THI5 are similar. The index values of THI1, THI3, and THI5 in the air inlet area are lower than the THI2 value. The difference between THI8 and BGHI distributions is not clear. Both animal occupied zone and animal non-occupied zone have a high THI6 index. For ET and ETIS, as they include the influence of air velocity, they have better performances than other indices. The maximum values of all contours using different thermal indices are a temperature of 34 °C, a relative humidity of 60%, and an air speed of 0 m·s^−1^, and the minimum values are a temperature of 30 °C, a relative humidity of 60%, and an air speed of 1 m·s^−1^, while the sow is at 38 °C. In the contours of H, the color around the sow is lighter, indicating that the H index cannot conspicuously display the thermal conditions around the sow.

Table 3 shows the correlation between the heat index and the heat dissipation of sows. ET and ETIS have the strongest correlation with the heat dissipation of sow among those analyzed indices. The THI2, THI4, and THI7 performs better than other THI indices, and these three indices have coefficients of determination higher than 0.8349. The THI1, THI3, and THI5 are significantly correlated with the heat transfer of the sow, and R^2^ is at least 0.826. THI1, THI3, and THI5 perform relatively poorly in predicting the heat exchange of a sow compared with THI2, THI4, and THI7. The correlation between H and THI6 and the heat transfer of sows are 0.8235 and 0.8189, respectively. However, THI8 and BGHI performed the worst in predicting heat transfer of sows.

## 4. Discussion

### 4.1. Comparison Using Equivalent Temperature Change Method

In a hot environment, an increase in air temperature will cause the sow’s respiration rate to increase, which removes a large amount of latent heat. The increase in humidity negatively impacts the latent heat dissipation, and the increase in relative humidity will increase the heat stress in sows. However, THI6 shows a downward trend as the relative humidity increases, which is inconsistent with reality. Previous studies have shown that at an air temperature of 30 °C, a 40% increase in relative humidity is equivalent to an increase of 1.9 °C in air temperature [52]. When H, THI8, and BGHI are at an air temperature of 30 °C, the change of 10% relative humidity can cause an increase in temperature of more than 1.73 °C, which is inconsistent with previous research. Previous studies have shown that when the temperature is higher than 32 °C, the possibility of cardiac failure in sows increases [6,53]. Studies have also shown that 32 °C is the upper critical ambient temperature for sows [54]. When ETIS is between 30 °C and 35 °C, the equivalent temperature changes drastically. When the air temperature is between 35 °C and 40 °C, as the relative humidity increases, the equivalent temperature of ETIS changes the most. The equivalent temperature performance of ETIS is more in line with previous studies [6,53]. Other indices did not show this effect. Therefore, compared with other heat indices, ETIS is more appropriate in predicting heat stress in sows.

### 4.2. The Effect of Air Velocity

When the air velocity is 0.4 m∙s^−1^–0.5 m∙s^−1^, the ET_25 °C_ curve and ET_winter_ curve intersect, which means the values of ET_25 °C_ and ET_winter_ are the same, but the skin surface feelings under 25 °C and in winter are different. This also applies to the ET_30 °C_ curve, which intersects with the ET_winter_ curve at an air velocity of 1.1 m∙s^−1^–1.2 m∙s^−1^. In summer, the ETIS won’t decrease as much as the ET does when the air velocity increases. When the air temperature is 40 °C, ET decreases as the air velocity increases. An air temperature of 40 °C is already higher than the core temperature of a sow. Based on the theory of heat transfer [55,56], when the air temperature is higher than the object temperature, the object is in a heated state, and increasing the airflow speed will cause more convective heat transfer from ambient air to the pig body. When the air temperature is 40 °C, ETIS increases as the air velocity increases. When taking the effect of air velocity into account, the ETIS is more appropriate than ET for predicting heat stress in sows.

### 4.3. Comparison Using Psychrometric Chart

In the psychrometric chart, the iso-line of THI6 under high temperature and high humidity is consistent with low temperature and low humidity, which obviously does not comport with reality. The main reason is that the relative humidity term in THI6 is a decreasing function, and the air temperature term is an increasing function. In order to keep the value of THI6 unchanged, it is necessary to increase the air temperature while increasing the relative humidity. For H, THI8, and BGHI, in the case of low relative humidity, higher air temperature can ensure that the heat index value is consistent with the heat index value of the air temperature of 22 °C and the relative humidity of 70%. This is also inconsistent with reality, because the temperature difference between 31.6 °C and 22 °C will be felt by animals. Assuming that the THI2 is constant, when the relative humidity changes from 0% to 70%, the air temperature will change from 22 °C to 28.4 °C. The change in air temperature is 6.4 °C, which can be felt by the sow. The temperature difference of the iso-ETIS line under different relative humidities is small, so ETIS neither completely depends on the air temperature, nor does it amplify the influence of relative humidity.

### 4.4. Comparison Using Computational Fluid Dynamics Method

ET and ETIS have the best predictions of environmental distribution and convective heat transfer of sows, mainly because ET and ETIS include the parameter of air velocity. As CFD mainly analyzes the impact of airflow on sows, the air velocity has a greater impact on the sow’s convective heat transfer coefficient than other parameters. H and THI include the influence of air temperature and relative humidity, but ignore the influence of air velocity. Therefore, the predictive effect of THI and H is lower than that of ET and ETIS. Due to different ratios and combinations of related parameters of air temperature and relative humidity, different THIs have slightly different prediction effects on environmental distribution. In addition, BGHI performs poorly in prediction, mainly because BGHI is the ratio of various parameters and considers radiation, but the CFD analysis does not consider the influence of radiation heat transfer.

### 4.5. Analysis of Existing Problems and Future Prospects

The ETIS performed better in the above-mentioned comparisons than other indices. This result is consistent with the result of Part 1 of the paper series [20]. It indicates that in the process of establishing the heat index, it is extremely important to select the heat transfer characteristics of sows as the main reference. The best performing THIs were THI2, THI4, and THI7, but this type of index does not include the impact of important factors such as air velocity. Although ET also performed well in the CFD analysis, ET showed unreasonable changes with the air velocity changing.

Although ETIS performs well in the comparison using various methods, it still has the following problems: (1) The heat transfer characteristics of pigs at different stages are different. Large pigs are afraid of heat and small pigs are afraid of cold. Different sizes and shapes will have different characteristic lengths [20,56,57]. Therefore, the ETIS threshold should be adjusted according to the growth stage of the pig. (2) Animals have different adaptability to different environments. In different climate zones, different species of animals have different comfort requirements for the thermal environment [57]. Different regions on the earth have different climatic characteristics. Therefore, ETIS thresholds in different regions will be different.

## 5. Conclusions

The following conclusions have been drawn:

1. It is better to use ETIS to analyze the heat stress in sows than using other thermal indices.
2. When the sow house only has temperature and humidity sensors and lacks air speed data, THI2, THI4 and THI7 can also be used to evaluate the sow’s thermal comfort index.
3. The threshold division of each index still needs to be improved, and the thermal stress threshold is an important parameter for evaluating the degree of heat stress. However, the heat dissipation characteristics of sows at different stages are different, and in different climate zones, different breeds of animal have different comfort requirements for the thermal environment. Therefore, threshold zones should be established according to the characteristics of sows at different stages and different climatic conditions.

## Figures and Tables

**Table 1:** Various thermal indices. Because there are many types of THI, a suffix of 1–8 is added for distinction. Among the various thermal indices, only some have threshold divisions.

Index	Calculation Equation	Threshold under Different Heat Stress Levels	Year
THI1	$THI1=T+0.36\cdot T_{wb}+41.5$	Thermal comfort: 61 < THI1 ≤ 65, Intermediate: 65 < THI1 ≤ 69, Thermal stress: 69 < THI1 ≤ 73	1958
THI2	$THI2=0.8\cdot T+\left(\frac{RH\cdot \left(T-14.4\right)}{100}\right)+46.4$	Suitable: THI2 < 74, Mild: 74 ≤ THI2 < 78, Moderate: 78 ≤ THI2 < 82, Severe: THI2 ≥ 82	1959
THI3	$THI3=0.65\cdot T+0.35\cdot T_{wb}$	Heat stress: THI2 ≥ 28	1965
THI4	THI4=T°−(0.55−0.0055·RH)·(T°−58)	-	1971
THI5	$THI5=0.72\cdot T+0.72\cdot T_{wb}+40.6$	Moderate: 75 ≤ THI5 < 78. Severe: THI5 ≥ 83	1972
THI6	$THI6=\left(1.8\cdot T+32\right)-\left(0.55\cdot \left(\frac{RH}{100}\right)\right)\cdot \left(\left(1.8\cdot T+32\right)-58\right)$	Suitable: THI6 ≤ 74, Mild: 74 < THI6 ≤ 78, Moderate: 78 < THI6 ≤ 84, Severe: THI6 > 84	1976
THI7	THI7=T−(0.55−0.0055·RH)·(T−14.5)	-	1976
THI8	$THI8=0.27\cdot T+1.35\cdot T_{wb}+34.07$	-	1983
BGHI	$BGHI=T_{g}+0.36\cdot T_{dp}+41.5$	-	1981
ET	$\begin{array}{ll} ET=T+0.0015\cdot & \left(RH-50\right)\cdot T\\ & +\left(-1.0\cdot 42-T\cdot \left(v^{0.66}-0.2^{0.66}\right)\right) \end{array}$	-	2018
ETIS	$\begin{array}{ll} ETIS=T+0.0006 & \cdot \left(RH-50\right)\cdot T-0.3132\\ & \cdot u^{0.6827}\cdot \left(38-T\right)-4.79\\ & \cdot \left(1.0086\cdot 38-T\right)+4.8957\\ & \cdot 10^{-8}\\ & \cdot ({\left(38+273.15\right)}^{4}\\ & -{\left(T+273.15\right)}^{4}) \end{array}$	Suitable: ETIS < 33.1, Mild: 33.1 ≤ ETIS < 34.5, Moderate: 34.5 ≤ ETIS < 35.9, Severe: 35.9 ≤ ETIS	2021
H	H=1.006·T+RHPm·10(7.5·T273.3+T)·(71.28+0.052·T)	-	2011

Note: T is the dry bulb temperature (°C); T° is the dry bulb temperature (°F); T_g_ is the black globe temperature (°C); T_wb_ is the wet bulb temperature (°C); T_dp_ is the dew point temperature (°C); RH is the relative humidity (%); H is the enthalpy (kJ·kg^−1^); and P_m_ is high mercury of barometric pressure (mmHg). THI is the temperature and humidity index. The numbers 1-8 after THI represent different forms of THI. BGHI is black globe-humidity index. ET is effective temperature, ETIS is equivalent temperature index of sows. H is enthalpy.

**Table 2:** Boundary conditions of each surface in Computational fluid dynamics simulation.

Boundary	Boundary Condition
Outlet	Velocity outlet, air velocity is 1 m·s^−1^
Inlet	Pressure outlet, the temperature is 30 °C relative humidity is 60%
Sow	No slip wall, temperature is 38 °C
Other (walls)	No slip wall, heat flux = 0

**Table 3:** The Coefficient of Determination (R^2^) between the sow’s heat transfer and heat index.

Heat Index	The Coefficient of Determination (R^2^)
THI1	0.8261
THI2	0.8351
THI3	0.8264
THI4	0.8349
THI5	0.8267
THI6	0.8189
THI7	0.835
THI8	0.7575
BGHI	0.8005
ET	0.9883
ETIS	0.9863
H	0.8235

Note: THI is the temperature and humidity index. The numbers 1-8 after THI represent different forms of THI, which are shown in Table 1. BGHI is black globe-humidity index. Et is effective temperature, ETIS is equivalent temperature index of sows. H is enthalpy.

## Data Availability

All data sets during the current study are available from the corresponding author on fair request.
